# Prognostic significance and immune landscape of a fatty acid metabolism-related gene signature in colon adenocarcinoma

**DOI:** 10.3389/fgene.2022.996625

**Published:** 2022-12-09

**Authors:** Xia Liu, Xisheng Fang, Lin Lu, Guolong Liu

**Affiliations:** Department of Medical Oncology, Guangzhou First People’s Hospital, South China University of Technology, Guangzhou, Guangdong, China

**Keywords:** colon adenocarcinoma, fatty acid metabolism gene, prognosis, risk signature, immune microenvironment

## Abstract

**Background:** Fatty acid metabolism (FAM), as a hallmark of caner, plays important roles in tumor initiation and carcinogenesis. However, the significance of fatty acid metabolism-related genes in colon adenocarcinoma (COAD) are largely unknown.

**Methods:** RNA sequencing data and clinical information were downloaded from the Cancer Genome Atlas (TCGA) cohort. Univariate and multivariate Cox regression analyses were utilized to construct a fatty acid metabolism-related gene signature. Kaplan-Meier survival and receiver operating characteristic (ROC) analyses were used to verify the performance of this signature. GEO datasets were applied to validate the signature. Maftools package was utilized to analyze the mutation profiles of this signature. Correlation between the risk signature and stemness scores was compared by RNA stemness score (RNAss). Gene Ontology (GO), Kyoto Encyclopedia of Genes and Genomes (KEGG) and gene set variation analysis (GSVA) were performed to explore the potential functions and signaling pathways. Immune landscape of the signature was explored by analyzing different immune cells infiltration, immune functions and microsatellite instability. A nomogram was constructed by combining the risk signature and multiple clinical factors. Expression levels and prognostic values of the risk genes were revealed in the cancer genome atlas and GEO databases. Moreover, the expression the risk genes were measured in cell lines using real time quantitative PCR (qRT-PCR).

**Results:** Eight fatty acid metabolism-related genes (CD36, ENO3, MORC2, PTGR1, SUCLG2, ELOVL3, ELOVL6 and CPT2) were used to construct a risk signature. This signature demonstrated better prognostic value than other clinicopathological parameters, with AUC value was 0.734 according to the cancer genome atlas database. There was negative correlation between the riskscore and RNA stemness score. The patients in the high-risk group demonstrated higher infiltration of M0 macrophages, and less infiltration of activated CD4 memory T cells and Eosinophils. There were more MSI patients in the high-risk group than those in the low-risk group (38% vs. 30%). The risk scores of patients in the MSI group were slightly higher than those in the microsatellite stability group. Gene ontology, kyoto encyclopedia of genes and genomes and gene set variation analysis enrichment analyses showed that several metabolism-related functions and signaling pathways were enriched. A nomogram showed good predictive capability of the signature. Moreover, qRT-PCR revealed upregulated expression of ENO3, MORC2, SUCLG2 and ELOVL6, and downregulated expression of CPT2 in all examined colon adenocarcinoma cell lines.

**Conclusion:** This study provided novel insights into a fatty acid metabolism-related signature in the prognosis an immune landscape of colon adenocarcinoma patients.

## Introduction

Colorectal cancer is the third most common malignancy and second predominant cause of cancer-related death worldwide (Sung et al., 2021). Among all the histopathological types, colon adenocarcinoma (COAD) accounts for the major part. The incidence of COAD is increasing yearly in China. Recently, the development of targeted therapy and immune checkpoint inhibitors (ICIs) have gained advancements in COAD (Loupakis et al., 2014; Chen 2015; Yaghoubi et al., 2019). However, only partial patients could benefit from these treatments, which depending on the gene mutation state and microsatellite instability status of cancer patients (Ganesh et al., 2019; Yaghoubi et al., 2019; Ladabaum et al., 2020). Therefore, it is essential to identify novel diagnostic and prognostic biomarker, as well as treatment target for COAD patients.

Metabolism reprogramming, as a hallmark of cancer, contributes to the carcinogenesis and progression of malignancies (Navarro et al., 2022; Zhang et al., 2022). Most of the current studies were focused on glycolysis and mitochondrial oxidative phosphorylation (Song et al., 2021; Jones et al., 2022). Recently, the roles of fatty acid metabolism (FAM) in tumor development have gained a lot of attentions (Wang et al., 2022; Xiong et al., 2022). Fatty acid metabolism could provide nutrition to support the growth of tumor cells and help tumor cells to adapt to tumor microenvironment (Zhang et al., 2022). Numerous studies have shown that abnormal expression of FAM-related genes was capable of predicting the survival of colon adenocarcinoma (Geng et al., 2021; Mokhtari et al., 2022). And FAM-related genes were involved in cell proliferation, migration and invasion (Liu Y. et al., 2022). For example, EPHX2 was identified as a tumor suppressor in colorectal carcinoma (CRC), which repressed CRC progression by inducing fatty acid degradation (Zhou et al., 2022). HMG-CoA lyase (HMGCL), which converts HMG-CoA to acetyl-CoA and a ketone body, could activate MEK1, and promote the proliferation of BRAF-positive melanoma and colon cancer cells (Zhao et al., 2017). HMGCL was reported as a prognostic factor in colon adenocarcinoma (Zhao F. et al., 2021). Although the predictive capabilities and functions of some FAM-related genes have been revealed, limited studies have comprehensively revealed the FAM-related gene signature in COAD. Moreover, the biological function and immune profile of these FAM-related genes were not well explored in COAD.

This study was designed to construct a predictive signature by comprehensively screening fatty acid metabolism-related genes. Moreover, the predictive capability, association with immune microenvironment, and functional enrichment of this risk signature were further studied. All these results suggested that this FAM-related gene signature with promising predictive capability in colon cancer. And this study would help us to further understand the mechanisms of FAM in COAD.

## Material and methods

### Data acquisition and identification of fatty acid metabolism-related genes

RNA sequencing data and clinical data of colon adenocarcinoma patients were obtained from the TCGA or GEO databases. There were 39 cases of normal tissues and 404 cases of COAD tumor tissues in TCGA-COAD cohort. Other datasets, including GSE39582 and GSE110224, were downloaded from the GEO database. GSE39582, including 585 COAD cases, was used for survival analyses. And GSE110224, including 17 pairs of normal tissues and tumor tissues, was utilized for differential expression analyses. A list of 309 FAM-related genes was acquired by literatures searching. Differentially expressed FAM-related genes were identified using “limma” package based on the following criteria |log2FC| > 1 and false discovery rate (FDR) < 0.05, and the results were visualized by a heatmap in R software (version 4.2.0).

### Univariate and multivariate cox regression analyses

FAM-related genes with prognostic values were identified using “survival” and “survminer” packages in R software. Firstly, univariate cox regression was utilized to retrieve prognostic FAM-related genes. Moreover, multivariate cox regression was performed to screen genes to construct a risk signature.

### The least absolute shrinkage and selection operator (LASSO) analysis

LASSO analysis was performed using “glmnet” and “survival” packages based on the survival data and expression data of the genes in the risk signature by using R software.

### Validation of the predictive capability of the risk signature in the cancer genome atlas-colon adenocarcinoma cohort

Risk score was calculated using the following formula: risk score = (coefficient1* mRNA expression level of gene1) + (coefficient2* mRNA expression level of gene2) +…… + (coefficientN* mRNA expression level of geneN). Patients were divided into high-risk group and low-risk group based on the median risk score. Firstly, Kaplan-Meier survival analysis was used to verify the performance of the risk signature in predicting the overall survival (OS) of cancer patients using “survival” package. ROC was conducted using “survivalROC” package. Risk score distribution and survival status distribution were visualized using R software.

### Validation of prognostic significance of risk model in GEO database

To validate the prominent prognostic value of the risk score, the gene expression microarray database GSE39582 was selected to perform further analysis. The risk score was calculated based on the expression of 8 FAM-related genes, followed by the Kaplan-Meier survival analysis and ROC analysis. Risk score and survival status distribution were also visualized by the “survival” package of R software.

### Mutation profiles of the risk signature

Somatic variation of the risk signature was profiled using “maftools” package in R software.

### Correlation with RNA stemness score (RNAss)

The correlation between the risk scores and RNA stemness score were compared in R software based on “StemnessScores_RNAexp_20170127.2. tsv”.

### Immune correlations

The immune cells infiltration data was retrieved from CIBERSORT tool. Immune cells infiltration data was retrieved using “e1071”, “parallel”, and “preprocessCore'” packages. The correlations between the risk signature and immune cells infiltration were assessed using “limma”, “reshape2” and “ggpubr” packages in R software. The differences in various immune functions between high-risk group and low-risk group were compared using “limma”, “GSVA”, “GSEABase”, “ggpubr” and “reshape2” packages in R software.

### Association between the risk score and microsatellite instability status

The correlations between risk score and MSI status were analyzed using “plyr”, “ggplot2” and “ggpubr” packages in R software.

### Functional enrichment analysis

The Gene Ontology (GO) and Kyoto Encyclopedia of Genes and Genomes (KEGG) functional enrichment analyses were used to explore potential biological functions and signal pathways using “clusterProfiler”, “org.Hs.eg.db”, “enrichplot”, and “ggplot2d” packages in R software.

### Gene set variation analysis (GSVA)

To explore pathways differentially enriched in high-risk and low-risk groups, GSVA was performed using “limma”, “GSEABase” and “GSVA” packages by using “c2. cp.kegg.v7.4. symbols.gmt” as a reference gene set. The results were visualized by a heatmap.

### Prognostic values of the risk signature

Univariate and multivariate cox regression analyses were utilized to determine the predictive capabilities of the FAM-related gene signature and various clinicopathological parameters using “survival” package in R software. Moreover, ROC of FAM-related gene signature and various clinicopathological parameters were compared using “survial”, “survminer” and “time ROC” packages.

### Construction of a nomogram

A nomogram including the risk signature and various clinical factors (age, gender and stage) was constructed using “survival”, “survminer”, and “timeROC” packages. The predictive capability of the nomogram was assessed by ROC curves. Univariate cox regression was utilized to explore the prognostic roles of the nomogram and various clinical parameters.

### Differential expression of the fatty acid metabolism-related genes in public database

RNA expression data of the 8 FAM-related genes were retrieved from the TCGA database and GEO dataset GSE110224. Firstly, the differential expression of these genes in colon tumor tissues and normal tissues were compared using unpaired *t* test according to data retrieved from the TCGA database. The differential expression of candidate genes retrieved from GEO database was identified using the “limma” and “ggpubr“ packages of R software (version 4.2.0).

### Cell line and cell culture

Human colonic epithelial cell line NCM460 and colon adenocarcinoma cells lines (HCT116, CT26 and SW480) were purchased from the Culture Collection of Chinese Academy of Sciences (Shanghai, China). And culture in medium. NCM460 was culture in DMEM medium. While HCT116, CT26 and SW480 were maintained in RPMI 1640 medium. All the culture medium was supplemented with 10% fetal bovine serum.

### Real time quantitative PCR (qRT-PCR)

Total RNA was extracted from various cell lines using TRIzol reagent (Thermo Fisher Scientific) as previously described (Lu et al., 2022). cDNA was synthesized and subjected to qRT-PCR. Gene expression was measured using ChamQ universal SYBR qPCR Master Mix. Relative gene expression was calculated using 2-△△Ct method. Relative expression of each gene was normalized to internal control GAPDH. The primer sequences were as follows: GAPDH Forward: CTC​CTC​CTG​TTC​GAC​AGT​CAG​C, Reverse: CCC​AAT​ACG​ACC​AAA​TCC​GTT; CD36 Forward: CTT​TGG​CTT​AAT​GAG​ACT​GGG​AC, Reverse: GCA​ACA​AAC​ATC​ACC​ACA​CCA; ENO3 Forward: TAT​CGC​AAT​GGG​AAG​TAC​GAT​CT, Reverse: AAG​CTC​TTA​TAC​AGC​TCT​CCG​A; MORC2 Forward: CAC​GTC​AAG​CCG​TTT​CAA​GAC, Reverse: AGG​CGT​ACT​TCT​AAT​GTC​CGA; PTGR1 Forward: AGC​ACT​TTG​TTG​GCT​ATC​CTA​C, Reverse: CCC​CAT​CAT​TGT​ATC​ACC​TTC​C; SUCLG2 Forward: CAA​AAG​ACC​CTA​ATG​TTG​TGG​GA, Reverse: TTC​AGC​AAC​CAT​CAC​CTT​GTT; ELOVL3 Forward: GGA​AGC​TGA​CAT​CCG​GGT​AG, Reverse: TCC​AGT​TCA​AGA​CAC​ACC​ACC; ELOVL6 Forward: GCA​CCC​GAA​CTA​GGA​GAT​ACA, Reverse: CCC​CGG​CAA​CCA​TGT​CTT​T; CPT2 Forward: CTG​GAG​CCA​GAA​GTG​TTC​CAC, Reverse: AGG​CAC​AAA​GCG​TAT​GAG​TCT.

### Survival analysis of the risk genes

Kaplan Meier survival analysis was utilized to explore the roles of the risk genes in predicting the overall survival of COAD patients. Survival data were retrieved from both TCGA-COAD cohort and GEO dataset GSE39582.

### Statistical analysis

All data analyses based on bioinformatic data were performed using R software (Version 4.0.2) or Perl software. Differential expression of the risk genes in various cell lines were compared using student *t* test in GraphPad Prism. A two-tailed *p* < 0.05 was statistical significance.

## Results

### Identification of prognostic fatty acid metabolism (FAM)-related gene signature in COAD

**FIGURE 1:**
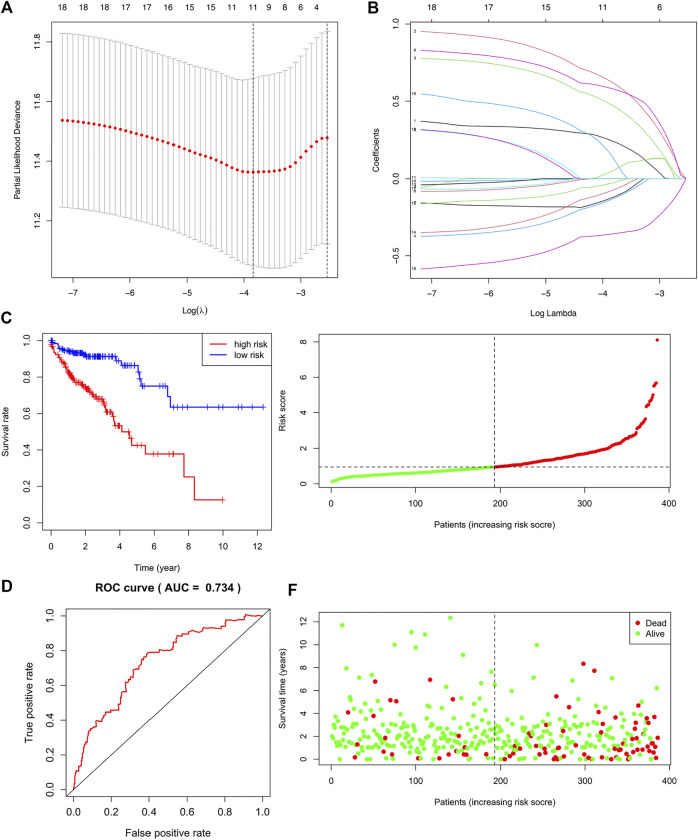
Construction and Validation of a fatty acid metabolism-related gene signature in COAD. **(A,B)** The LASSO coefficient profiles of the 18 FAM-related genes signature. **(C)** Kaplan-Meier (KM) analysis of OS based on the 8 FAM-related signature. **(D)** ROC analysis of the risk signature in predicting the OS. **(E)** Survival status distribution in high-risk group and low-risk group. **(F)** Risk score distribution of the high-risk group and low-risk group.

A list of 309 fatty acid metabolism (FAM)-related genes were acquired from literatures screening (Chang and Xing 2022; Li et al., 2022; Tang et al., 2022). RNA-sequencing data of COAD patients were downloaded from the TCGA-COAD cohort. Differentially expressed FAM-related genes were retrieved and displayed in a heatmap (Supplementary Figure S1). Univariate Cox regression analysis identified 18 FAM-related genes with prognostic values (Table 1). Lasso regression analysis was utilized to assess the 18 pronostic related genes (Figures 1A,B). Moreover, multivariate Cox regression analysis identified 8 genes to construct a risk model, which including CD36, ENO3, MORC2, PTGR1, SUCLG2, ELOVL3, ELOVL6 and CPT2 (Table 2).

**TABLE 1:** Univariate Cox regression analysis of FAM-related genes in COAD.

Gene	HR	HR.95L	HR.95H	P-value
CD36	1.480685977	1.119197563	1.958931143	0.005986258
ENO3	2.881938078	1.541099066	5.389379091	0.00091922
MORC2	2.986501011	1.514485104	5.889254552	0.00158828
PTGR1	0.628078483	0.431322459	0.914588548	0.015283413
SUCLG2	0.695783976	0.518216185	0.934195718	0.015832456
ELOVL3	2.421379226	1.425573808	4.112784143	0.001068716
ACOT11	0.499264429	0.285428979	0.873299449	0.014897922
GPX2	0.725075105	0.571708529	0.919583811	0.008016401
CIDEA	1.859825383	1.234231146	2.802514317	0.003017924
CA2	0.882891759	0.783978238	0.994285072	0.039927092
G0S2	1.211409361	1.009058542	1.454338454	0.039718061
HADH	0.652953437	0.445386672	0.957254039	0.028978383
EPHX2	0.768291156	0.601941354	0.980612641	0.03424123
ELOVL6	0.704043266	0.498017959	0.99529929	0.04696243
ACOX1	0.509350909	0.292564701	0.886772558	0.017090788
ACADL	4.211789449	1.548807683	11.45343645	0.004846323
HMGCL	0.508152821	0.304403686	0.848279116	0.009616349
CPT2	0.467689111	0.300575572	0.727714175	0.000754246

**TABLE 2:** Multivariate Cox regression analysis of FAM-related genes in COAD.

Gene	Coef	exp (coef)	se (coef)	z	Pr (>|z|)
CD36	1.264156393	3.54010502	0.462552575	2.733000443	0.006276025
ENO3	2.530859605	12.56430184	0.940955548	2.689669677	0.007152278
MORC2	3.153129423	23.40920739	1.612288117	1.955686078	0.050502146
PTGR1	−1.722078824	0.178694288	0.918573122	−1.874732434	0.060829543
SUCLG2	2.063616289	7.874394467	1.351187043	1.52726175	0.126695937
ELOVL3	2.318193907	10.15731268	0.782167858	2.963806148	0.003038596
ELOVL6	−1.116202638	0.327521153	0.748537668	−1.491177647	0.135914861
CPT2	−2.813370174	0.060002433	1.226134874	−2.294503022	0.021761622

### Validation of the fatty acid metabolism-related genes risk signature

Risk score was calculated based on the coefficient values and mRNA expression data of these eight risk genes. The patients were divided into high-risk and low-risk groups based on the median risk score. We firstly validated the performance of the risk signature in predicting the OS of colon adenocarcinoma patients. KM curve revealed that COAD patients in high-risk group demonstrated worse survival than patients in low-risk group (Figure 1C, *p* = 0.243e-08). The AUC of the ROC analysis was 0.734, which indicating that the signature was with good prognostic accuracy (Figure 1D). The survival status and risk sore distribution showed that the risk signature could well differentiate the high-risk and low-risk groups (Figures 1E,F). Moreover, we validated the risk signature using GEO database (Supplementary Figure S2).

### Mutation profiles and relationships with stemess scores of the risk model

**FIGURE 2:**
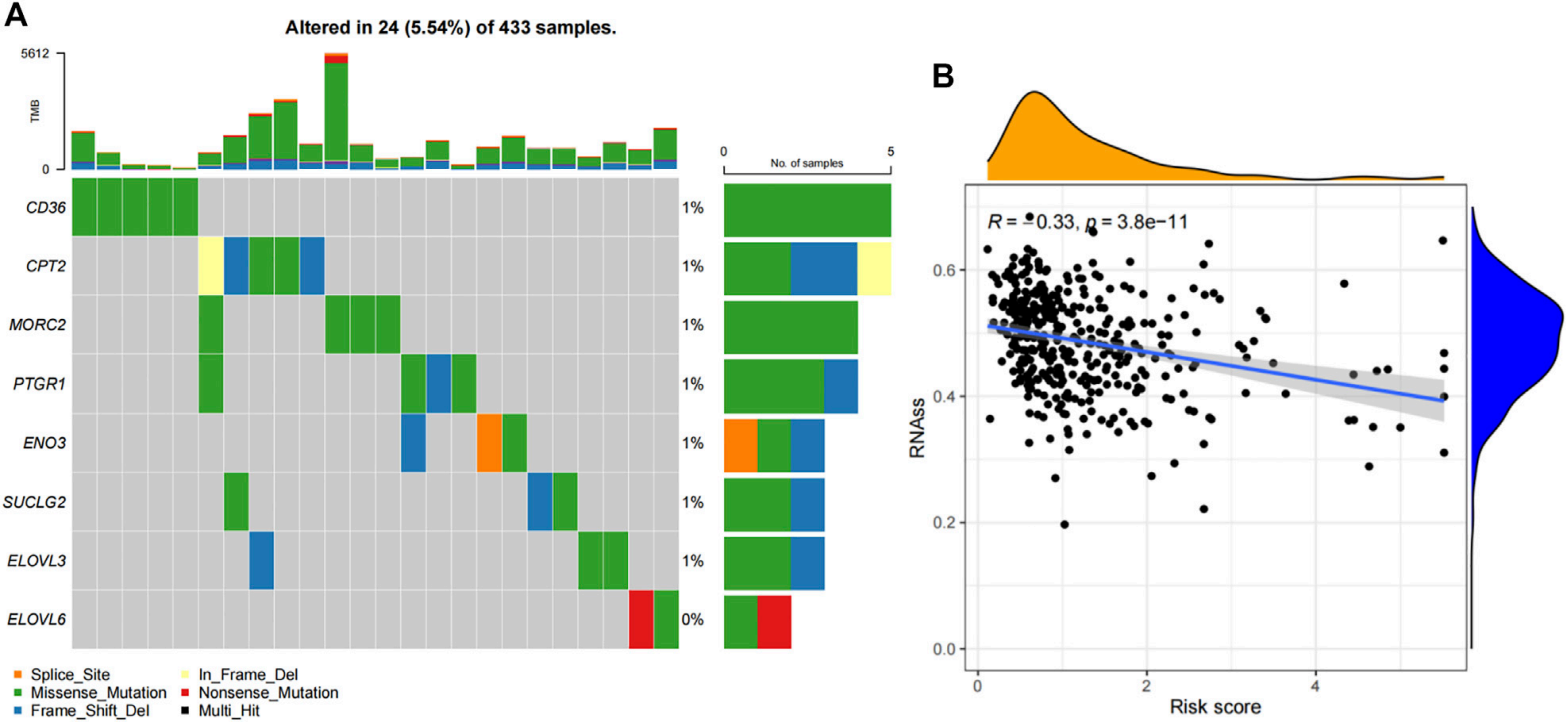
Mutation profiles and stemness correlation of the FAM-related signature. **(A)** The mutation profiles of these 8 genes. **(B)** The association between the risk score and RNAss.

The mutation frequencies of these risk genes were analyzed and displayed by a “maftools” package. As shown in Figure 2A, the mutation rates of these genes were very low. Moreover, there was negative correlation between the risk core and RNA stemness score (RNAss) (R = −0.33, *p* = 3.8e-11, Figure 2B).

### Immune landscape of the risk signature

**FIGURE 3:**
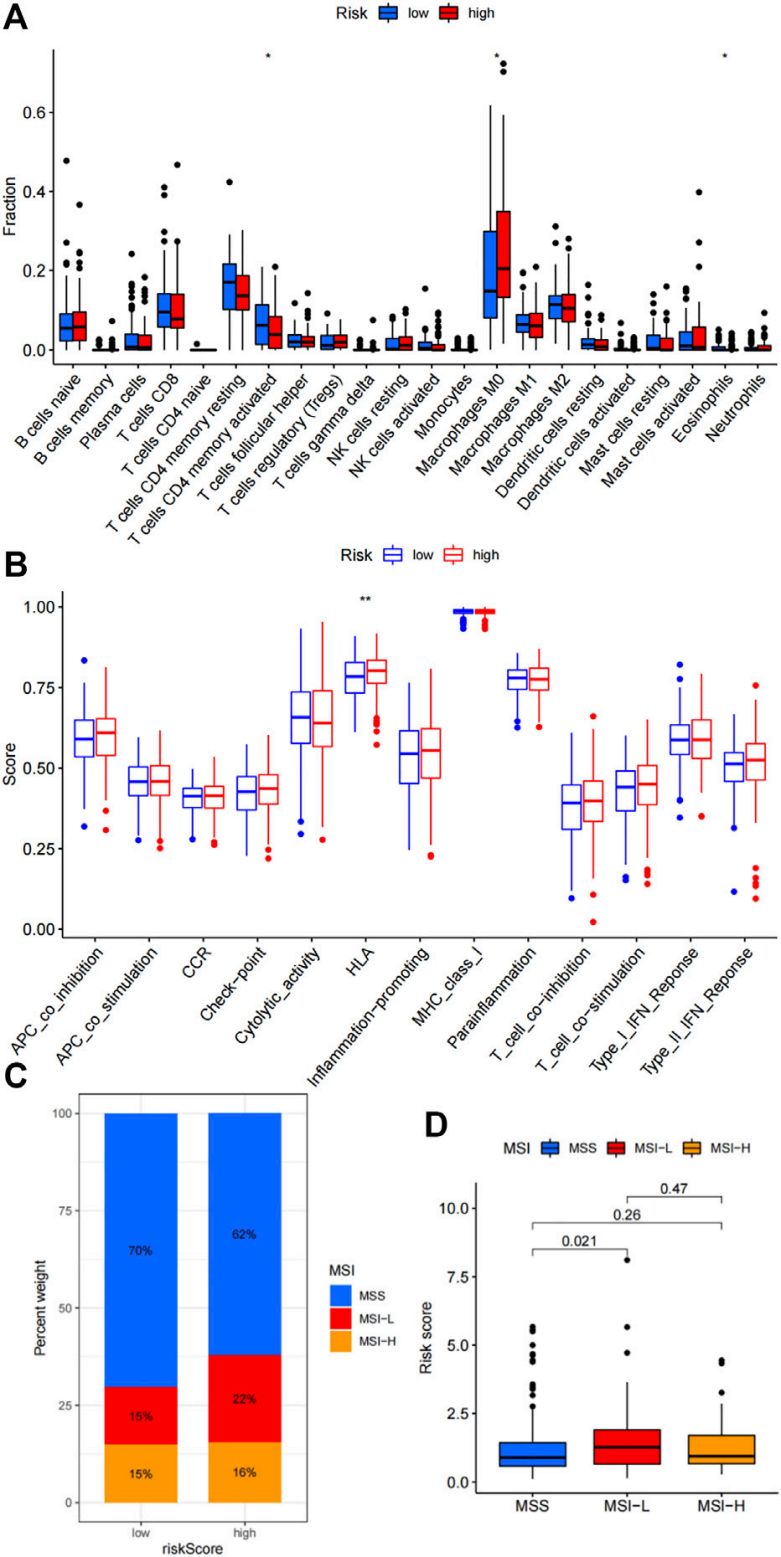
Association of the risk signature with immune microenvironment. **(A)** Differences in immune cells infiltration between high-risk and low-risk groups. **(B)** Correlations between the risk signature and various immune functions. **(C)** Percent weight of MSI status between high-risk and low-risk groups. **(D)** Comparison of risk scores among MSS, MSI-L and MSI-H subpopulation.

Given the immunotherapies have changed the treatment strategies of colon cancer, as well as there are limited effective biomarkers in predicting the treatment efficacy of immunotherapy, we further investigated the immune landscape of the risk signature. We firstly studied the associations between the risk signature and immune cells infiltration. High-risk group demonstrated more infiltration of M0 macrophages, and less infiltration of activated CD4+memory T cells and Eosinophils (Figure 3A, *p* < 0.05). Correlations between the risk signature and immune functions showed that high-risk group demonstrated upregulated HLA function (Figure 3B, *p* < 0.001). Microsatellite stability status was compared between high-risk and low-risk groups. As shown in Figure 3C, there were 70% patients with microsatellite stability (MSS), 15% patients with microsatellite instability-low (MSI-L) and 15% patients with microsatellite instability-high (MSI-H) in the low-risk group. While, 62% patients were MSS, 22% patients were MSI-L and 16% patients were MSI-H in the high-risk group. Moreover, the correlations between the risk score and microsatellite instability status were compared. We can see that the risk score of MSI-L were much higher than MSS (*p* = 0.021, Figure 3D).

### Functional enrichment analyses of the risk model

**FIGURE 4:**
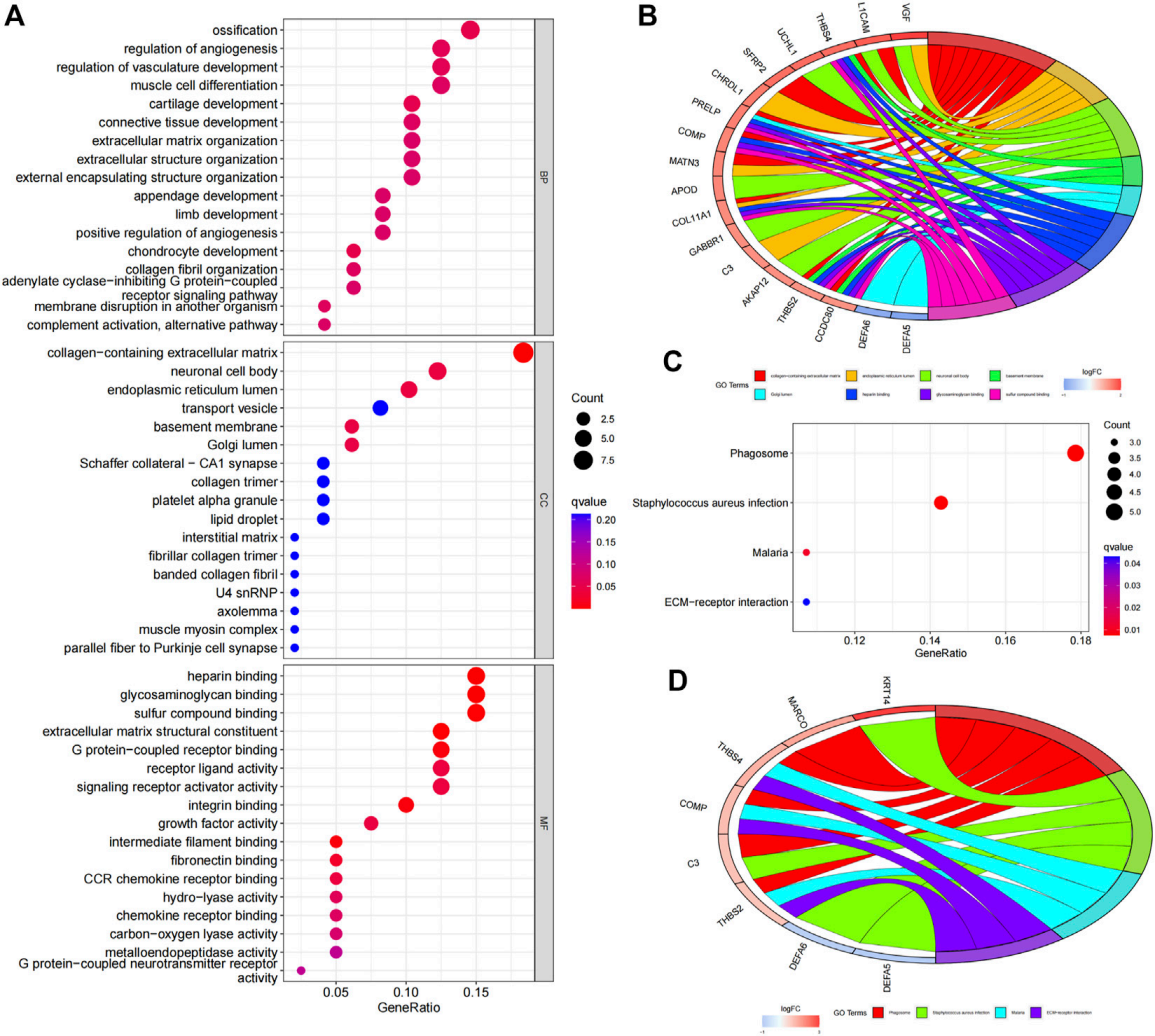
Functional enrichment analyses of the FAM-related gene signature. **(A)** GO analysis reveals biological process (BP), cellular components (CC), and molecular function (MF). **(B)** GOcircos of the genes enriched in different GO terms. **(C)** KEGG enrichment analysis of the risk signature. **(D)** KEGGcircos of the genes enriched in various KEGG pathways.

We further clarified the potential functions and mechanisms of the risk signature. GO enrichment analysis showed that the biological process (BP) was mainly enriched in “ossification”, “regulation of angiogenesis”, “regulation of vasculature development”, “muscle cell differentiation”, “cartilage development” *etc.* (Figure 4A). In the cellular components (CC), the risk signature was mainly enriched in “collagen-containing extracellular matrix”, “endoplasmic reticulum lumen”, “neuronal cell body”, “basement membrane”. As for molecular functions (MF), the risk model was mainly enriched in “heparin binding”, “glycosaminoglycan binding”, “sulfur compound binding”, “extracellular matrix structural constituent” (Figure 4A). GOcircos showed differentially expressed genes enriched in various GO terms (Figure 4B).

KEGG enrichment analysis showed that the underlying signal pathways were mainly enriched in “Phagosome”, “*Staphylococcus aureus* infection”, “Malaria” and “ECM-receptor interaction” (Figure 4C). KEGGcircos revealed differentially expressed genes enriched in different KEGG signaling pathway (Figure 4D).

**FIGURE 5:**
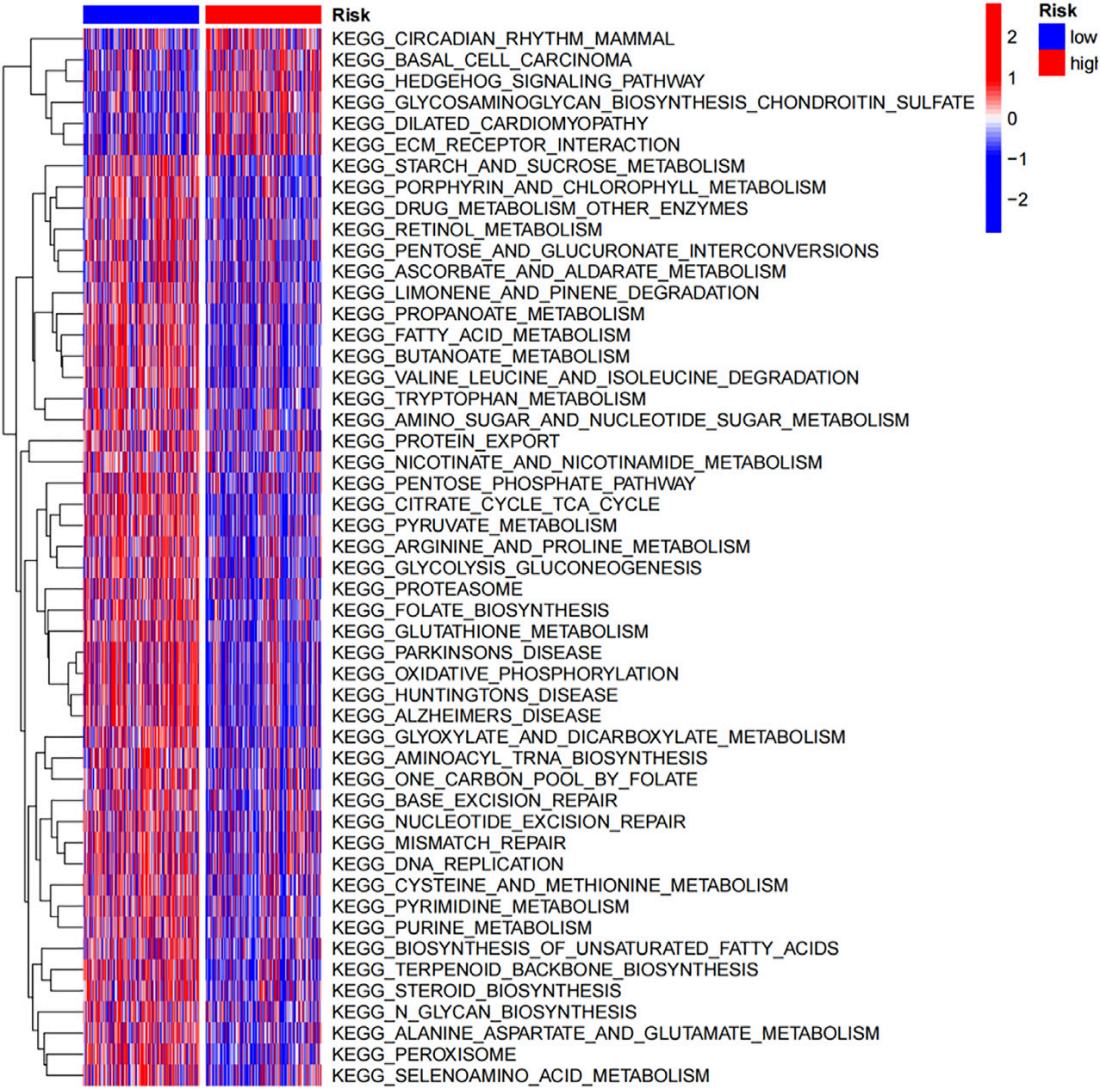
GSVA shows the potential signaling pathways enriched in high-risk and low-risk groups.

Moreover, a gene set variation analysis (GSVA) was performed to estimate underlying pathways. As shown in Figure 5, pathways, such as circadian rhythm mammal, basal cell carcinoma, Hedgehog signaling pathway, glycosaminoglycan biosynthesis chondroitin sulfate and ECM receptor interaction, were enriched in high-risk group. Signaling pathways, such as starch and sucrose metabolism, porphyrin and chlorophyll metabolism, drug metabolism other enzymes and retinol metabolism, were activated in low-risk group.

### Independent prognostic value of the risk signature

**FIGURE 6:**
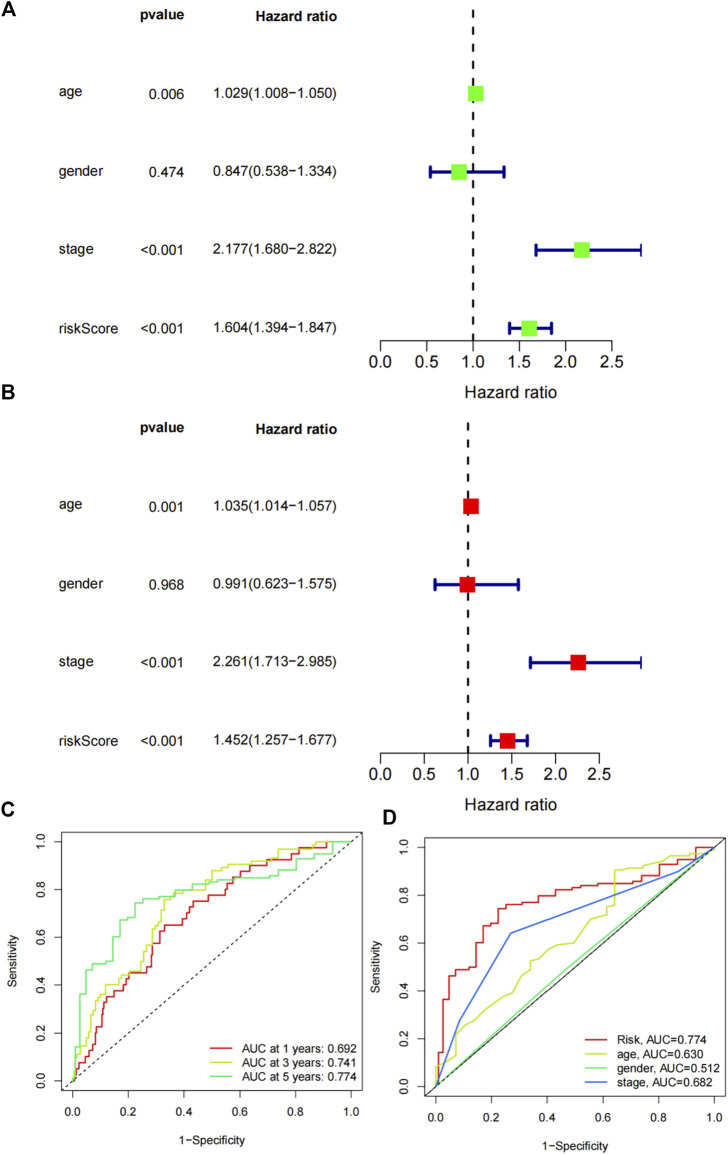
Predictive capabilities of the risk signature. **(A)** Univariate Cox regression analysis of the riskscore and various clinical factors. **(B)** Multivariate Cox regression analysis of the clinical factors and riskscore. **(C)** Time-dependent ROC curvesof the risk signature in predicting the survival. **(D)** Multi ROC curves of the risk signature and clinical parameters.

We further assessed the significances of the risk signature and common clinical factors. Univariate cox regression analysis indicated that age (*p* = 0.006, HR = 1.029, 95% CI:1.008-1.050), stage (*p* < 0.001, HR = 2.199 95% CI:1.680-2.822) and riskScore (*p* < 0.001, HR = 1.604, 95% CI:1.394-1.847) were prognostic factors for colon adenocarcinoma patients (Figure 6A). Further multivariate cox regression analysis demonstrated the independent predictive roles of age (*p* = 0.001, HR = 1.035, 95% CI:1.014-1.057), stage (*p* < 0.001, HR = 2.261 95% CI:1.713-2.985) and riskScore (*p* < 0.001, HR = 1.452, 95% CI:1.257-1.677) (Figure 6B). Time-dependent ROC curves showed that the AUC values of the signature in predicting 1 year, 3 years and 5 years survival were 0.692, 0.741 and 0.774, respectively (Figure 6C). Multi ROC curves comparing the Risk, age, gender and stage showed that the risk signature was better than other clinical parameters (AUC of risk: 0.774, Figure 6D).

### Establishment of a nomogram

**FIGURE 7:**
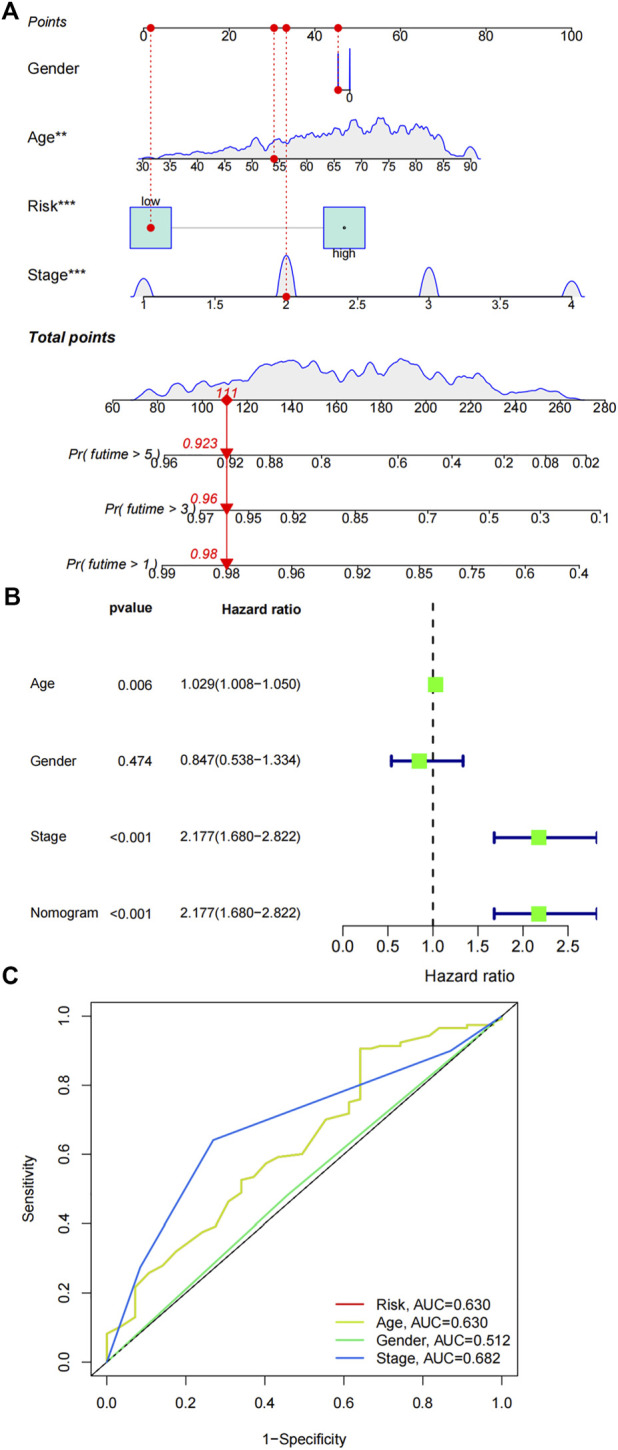
Construction of a nomogram. **(A)** A prognostic nomogram was constructed by combining risk signature, age, gender and stage. **(B)** Univariate cox regression analysis of the nomogram and various clinical parameters. **(C)** ROC results of the nomogram and various clinical parameters in predicting the survival of COAD patients.

The FAM-related gene signature and clinicopathological parameters (age, gender and stage) were combined to construct a prognostic nomogram. Age, stage and risk signature demonstrated prognostic values in the nomogram (Figure 7A). Univariate cox regression analysis further verified the predictive capabilities of age (*p* = 0.006, HR = 1.029 95% CI:1.008-1.050), stage (*p* < 0.001, HR = 2.177 95% CI:1.680-2.822) and nomogram (*p* < 0.001, HR = 2.177 95% CI:1.680-2.822) (Figure 7 B). Multi ROC curves comparing the nomogram and clinical factors showed that the AUC of nomogram was 0.63 (Figure 7C).

### Expression levels of the risk genes

**FIGURE 8:**
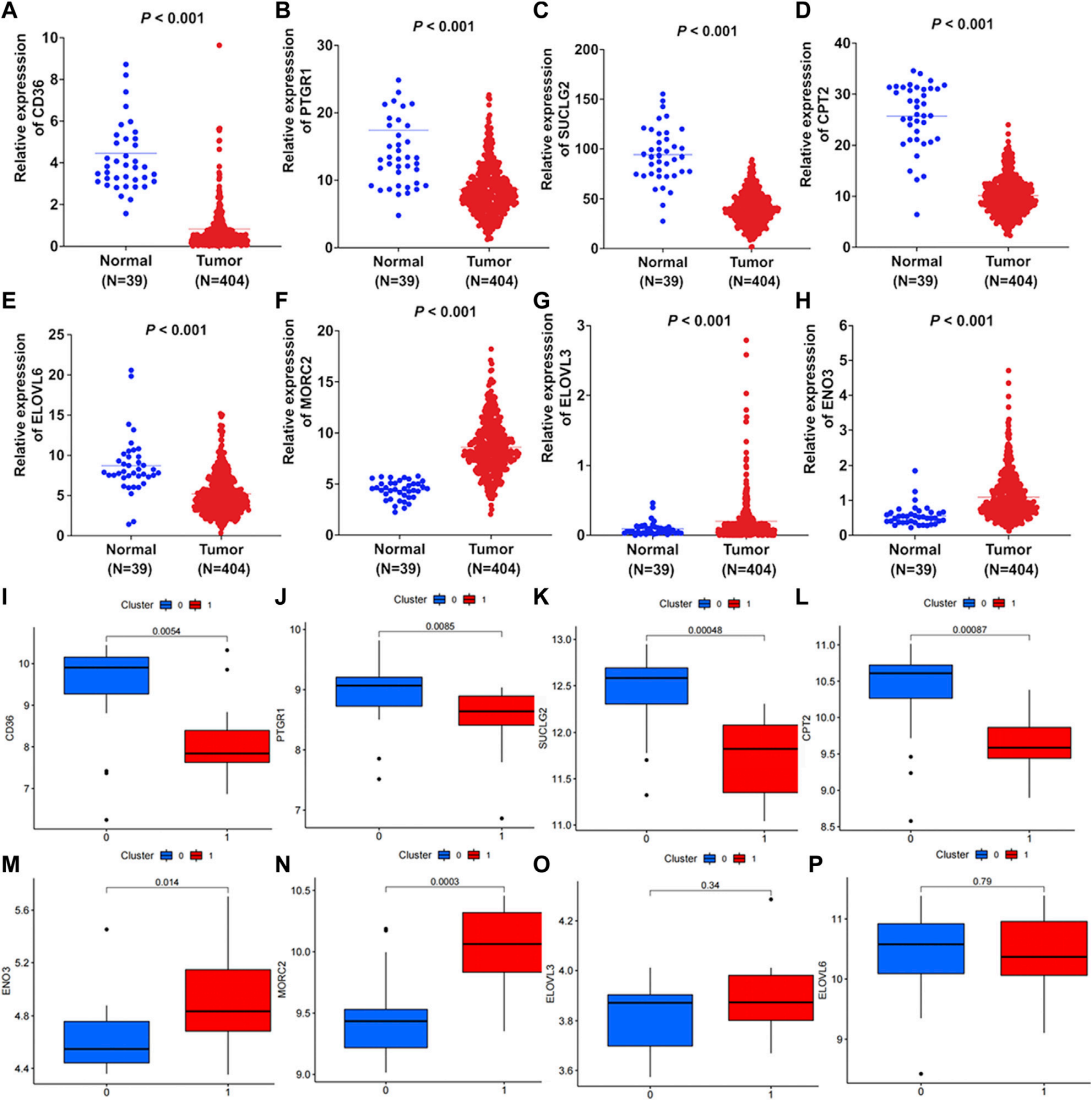
Differential expression of the FAM-related genes. Differential expression of **(A)** CD36, **(B)** PTGR1, **(C)** SUCLG2, **(D)** CPT2, **(E)** ELOVL6, **(F)** MORC2, **(G)** ELOVL3 and **(H)** ENO3 between colon adenocarcinoma tissues and normal tissues were compared according to TCGA-COAD cohort. GEO dataset validated the differential expression of **(I)** CD36, **(J)** PTGR1, **(K)** SUCLG2, **(L)** CPT2, **(M)** ENO3, **(N)** MORC2, **(O)** ELOVL3 and **(P)** ELOVL6 between tumor tissues and normal tissues. There were 17 pairs of normal tissues and tumor tissues in the GEO dataset.

Firstly, differential expression of these eight FAM-related genes were compared between colon adenocarcinoma tumor tissues and normal tissues in TCGA. As shown in Figures 8A–H, the expression of CD36, PTGR1, SUCLG2, CPT2 and ELOVL6 were downregulated in tumor tissues. While the expression of MORC2, ELOVL3 and ENO3 were upregulated in colon adenocarcinoma tumor tissues (Figures 8A–H, *p* < 0.001). Moreover, differential exression of these genes between normal tissues and cancer tissues were verified by GEO dataset. As shown in Figures 8I–L, the expression levels of CD36, PTGR1, SUCLG2 and CPT2 were significantly decreased in tumor tissues. And the expression levels of MORC2, ELOVL3 and ENO3 were significantly increased in tumor tissues (Figures 8M–O). However, there was no significant difference in the expression of ELOVL6 between normal tissues and tumor tissues (Figure 8P).

### Validation of the expression of the risk fatty acid metabolism-related genes in colon adenocarcinoma cell lines

**FIGURE 9:**
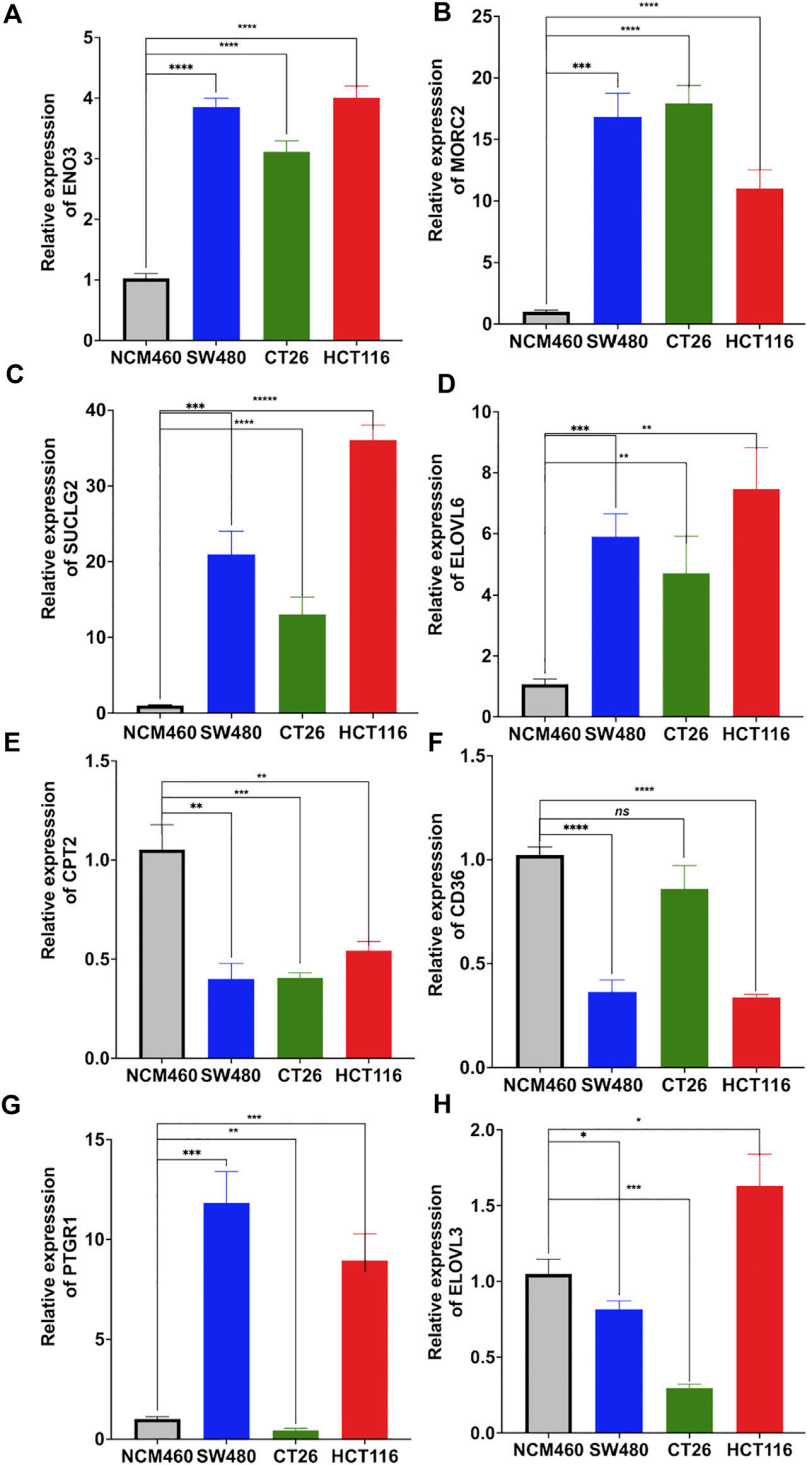
Relative mRNA expression of the risk signature genes. Real time quantitative PCR was utilized to compare the expression of ENO3 **(A)**, MORC2 **(B)**, SUCLG2 **(C)**, ELOVL6 **(D)**, CPT2 **(E)**, CD36 **(F)**, PTGR1 **(G)** and ELOVL3 **(H)** between human colon mucosal cell line (NCM460) and colon adenocarcinoma cell lines (SW480, CT26 and HCT116).

Moreover, expression levels of these 8 genes in colon adenocarcinoma cell lines were compared with human colon epithelial cell line by using real time quantitative PCR (qRT-PCR). Results showed increased expression of ENO3 (Figure 9A), MORC2 (Figure 9B), SUCLG2 (Figure 9C) and ELOVL6 (Figure 9D), and decreased expression of CPT2 (Figure 9E) in all examined colon adenocarcinoma cell lines. Compared to normal cell line NCM460, downregulated expression of CD36 was detected in SW480 and HCT116 (Figure 9F). The expression PTGR1 was upregulated in SW480 and HCT116, and downregulated in CT26 (Figure 9G). The expression of ELOVL3 was decreased in SW480 and CT26, and upregulated in HCT116 (Figure 9H).

### Survival analysis of the fatty acid metabolism-related risk genes

**FIGURE 10:**
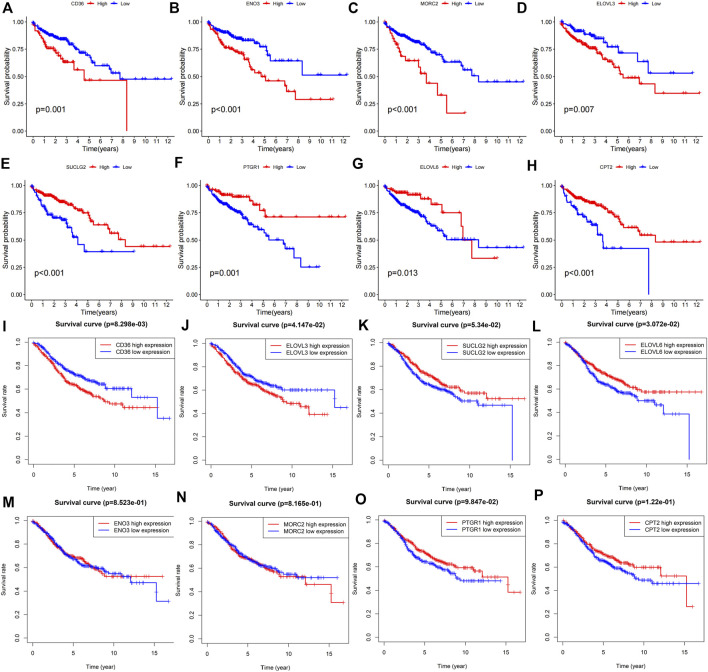
Prognostic roles of the risk FAM-related genes in COAD. Kaplan-Meier survival analyses were utilized to study the predictive roles of **(A)** CD36, **(B)** ENO3, **(C)** MORC2, **(D)** ELOVL3, **(E)** SUCLG2, **(F)** PTGR1, **(G)** ELOVL6 and **(H)** CPT2 according to TCGA-COAD cohort. GEO dataset validated the prognostic values of **(I)** CD36, **(J)** ELOVL3, **(K)** ELOVL6, **(L)** SUCLG2, **(M)** ENO3, **(N)** MORC2, **(O)** PTGR1 and **(P)** CPT2 in colon adenocarcinoma.

The Kaplan-Meier survival curves of these eight FAM-related genes were displayed in Figure 10. High expression of CD36 (Figure 10A, *p* = 0.001), ENO3 (Figure 10B, *p* < 0.001), MORC2 (Figure 10C, *p* < 0.001) and ELOVL3 (Figure 10D, *p* = 0.007) predicted poor overall survival in COAD patients. While low expression of SUCLG2 (Figure 10E, *p* < 0.001), PTGR1 (Figure 10F, *p* = 0.001), ELOVL6 (Figure 10G, *p* = 0.013) and CPT2 (Figure 10H, *p* < 0.001) were correlated with poor overall survival of colon adenocarcinoma patients. Moreover, the prognostic values of these genes were verified using GEO dataset. High expression of CD36 (Figure 10I, *p* = 8.298e-03) and ELOVL3 (Figure 10J, *p* = 4.147e-02) predicted poor OS, while high expression of SUCLG2 (Figure 10K, *p* = 3.072e-02) predicted better OS. There were no statistical significances of SUCLG2, ENO3, MORC2, PTGR1 and CPT2 in predicitng the OS of COAD patients (Figures 10L–P).

## Discussion

Fatty acid metabolism has been proven to contribute to multiple processes of cancer development, including tumor initiation, distant metastasis, recurrence and treatment failure (Cheng et al., 2022; Shueng et al., 2022; Tao et al., 2022; Wilcock et al., 2022). Dysregulated expression of FA-related genes were major contributors to the obstacle of fatty acid metabolism (Zhao et al., 2017; Liu et al., 2019; Park et al., 2019; Zhao F. et al., 2021; Zhou et al., 2022). Although there is lack of fatty acid metabolism therapy for the treatment of cancer patients, targeting FAM-related genes demonstrated promising roles in cancer treatment. Numerous studies have identified FAM-related signatures in various tumors (Liu et al., 2019; Park et al., 2019; Zhao F. et al., 2021). However, FAM-related gene signature in colon adenocarcinoma still remains explored thoroughly.

Prognostic signatures based on gene sets with various biological traits demonstrate better sensitivity and specificity than one single biomarker (Zhao F. et al., 2021). Thus, in this study, we comprehensively explored 309 FAM-related genes and selected genes with different expression and prognostic values to construct a risk signature. In this study, FAM-related genes (CD36, ENO3, MORC2, PTGR1, SUCLG2, ELOVL3, ELOVL6 and CPT2) were selected to construct a risk signature. Moreover, the expression, prognostic roles, potential functions and immune landscape of the FAM-related signature were comprehensively andsystemically verified.

The identified FAM-related risk signature in predicting the prognosis and TNM stage were validated, which displayed better capability. Aberrant expression of FA-related genes could predict the survival of colorectal cancer patients. Numerous studies have displayed the prognostic roles of CD36 in colorectal cancer (Pang et al., 2019; Wang XQ. et al., 2021; Zhao J. et al., 2021). CD36 demonstrated multiple signatures in CRC, such as immune-related and DNA damage and repair-related (Wang XQ. et al., 2021). Li et al. found that COMP phosphorylated ERK and Akt to upregulate EMT markers through CD36 signaling (Li et al., 2018). Inhibition of CD36 might prove beneficial in repressing metastasis spread, which demonstrated clinical practice potential (Enciu et al., 2018). ENO3, as a glycolysis and hypoxia-related gene, could predict the prognosis and immune landscape of colon cancer patients (Cui et al., 2021; He et al., 2021; Mao et al., 2022). ENO3 could promote CRC cell proliferation and migration by promoting glycolysis metabolism (Chen et al., 2022). Park et al. found that knockdown of ENO3 could selectively target mutant lung cancer (Park et al., 2019). MORC2 expression was widely upregulated in most cancer types, and aberrant MORC2 expression predicted the survival of cancer patients (Ding et al., 2018). Liu et al. revealed that MORC2 promoted the migration, invasion and metastasis of CRC by repressing NDRG1 (Liu et al., 2019). PTGR1, as an inducible enzyme with enone reductase activity, demonstrated therapeutical potential for cancer treatment (Yu et al., 2012; Wang X. et al., 2021). Upregulated expression of PTGR1 promoted cell proliferation and resistance to oxidative stress in hepatocellular carcinoma (Sánchez-Rodríguez et al., 2017). Upregulated expression of ELOVL3 was identified in hepatocellular carcinoma and colorectal cancer, which was a risk factor for these cancer patients (Alnabulsi et al., 2019; Yuan et al., 2021). CPT2 was once reported to predict the survival of CRC patients (Liu F. et al., 2022; Su et al., 2022). Liu et al. revealed that knockdown of CPT2 promoted proliferation of CRC cells (Liu F. et al., 2022). Although most of the risk genes demonstrated prognostic values, in this study the risk signature demonstrated better predictive capability than other clinical factors.

Moreover, a nomogram was established based on the FAM-related risk score and other clinical factors. The nomogram also indicated good performance in predicting the prognosis of colon adenocarcinoma patients. These risk signature offers a novel method to predict the survival of colon adenocarcinoma patients. Additionally, the functional enrichment analyses showed that the risk signature demonstrated multiple biological functions and signaling pathways, which needed further explored.

Immune landscape, as major components of tumor microenvironment, determines the survival and response to immunotherapy. Analysis of the correlations between the riskscore and immune cells infiltration showed that activated CD4 memory T cells and eosinophils were less infiltrated in high-risk group, while M0 macrophages infiltrated more in high-risk group. And the HLA function was enhanced in the high-risk group. These data indicated the high-risk group demonstrating immunosuppressive phenotype. Moreover, we found that patients in the high-risk group were with more microsatellite instability status. Microsatellite instability status is known as a molecular fingerprint that changes the treatment strategies for colon cancer patients (Dekker et al., 2019). MSI colon cancers were characterized by a better prognosis, less sensitivity to chemotherapy and better response to immune checkpoint inhibitors (Boland and Goel 2010). Approximately 10–25% colon patients are MSI(Cohen et al., 2017). These data indicated that the risk signature might partially predict potential response to chemotherapy or immunotherapy.

Furthermore, we verified the expression of the 8 prognostic FAM-related genes in pubic databases and colon cancer cell lines. Some risk genes didn’t demonstrate statistical differences in the expression and prognosis in GEO datasets. These discrepancies might be due to the different clinical samples. By analyzing TCGA database, we found that CPT2, ENO3 and MORC2 showed consisted results in tissue expression, cell line expression and survival analysis. However, it is noted that there are some limitations of this study. The expression and predictive capability of this risk model needed to be validated in clinical specimens. Moreover, the concrete roles and mechanisms of FAM-related genes, such as MORC2, ENO3 and CPT2, need to be further clarified.

In conclusion, our research provided novel insights into a FAM-related gene signature in predicting the survival and immune landscape of colon adenocarcinoma.

## Data Availability

Publicly available datasets were analyzed in this study. This data can be found at TCGA project (“https://portal.gdc.cancer.gov/”). Further inquiries of the analyzed data can be directed to the corresponding authors.
