# Supplementary material for: Prognostic significance and immune landscape of a fatty acid metabolism-related gene signature in colon adenocarcinoma
Source: Front Genet. 2022 Dec 9;13:996625. doi: 10.3389/fgene.2022.996625 (PMC9780302; doi:10.3389/fgene.2022.996625)
Supplement: Supplementary file 1 [file DataSheet1.docx]

Supplementary Material


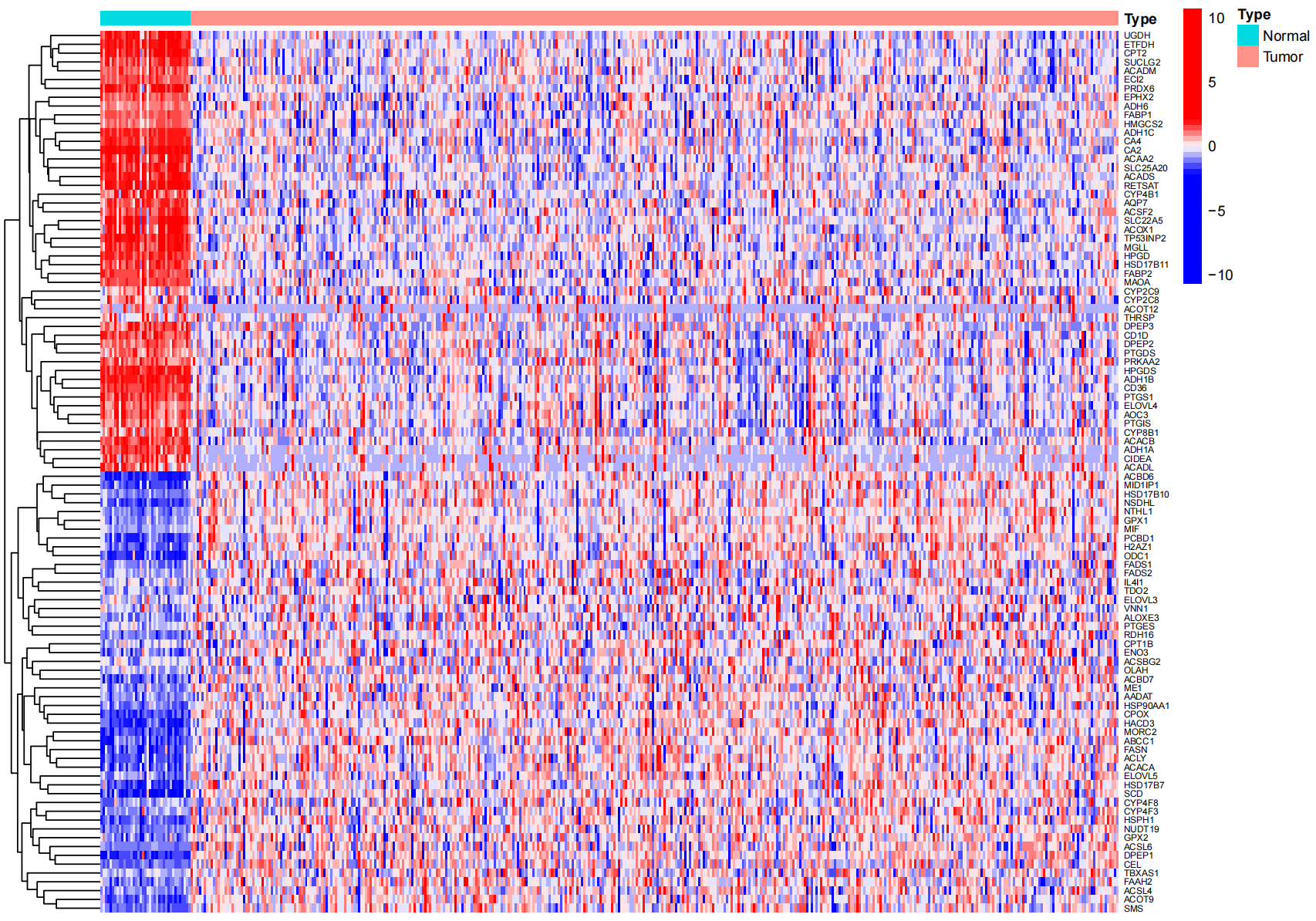


**Supplementary Figure 1**. Identification of differential expressed FAM-related genes in COAD


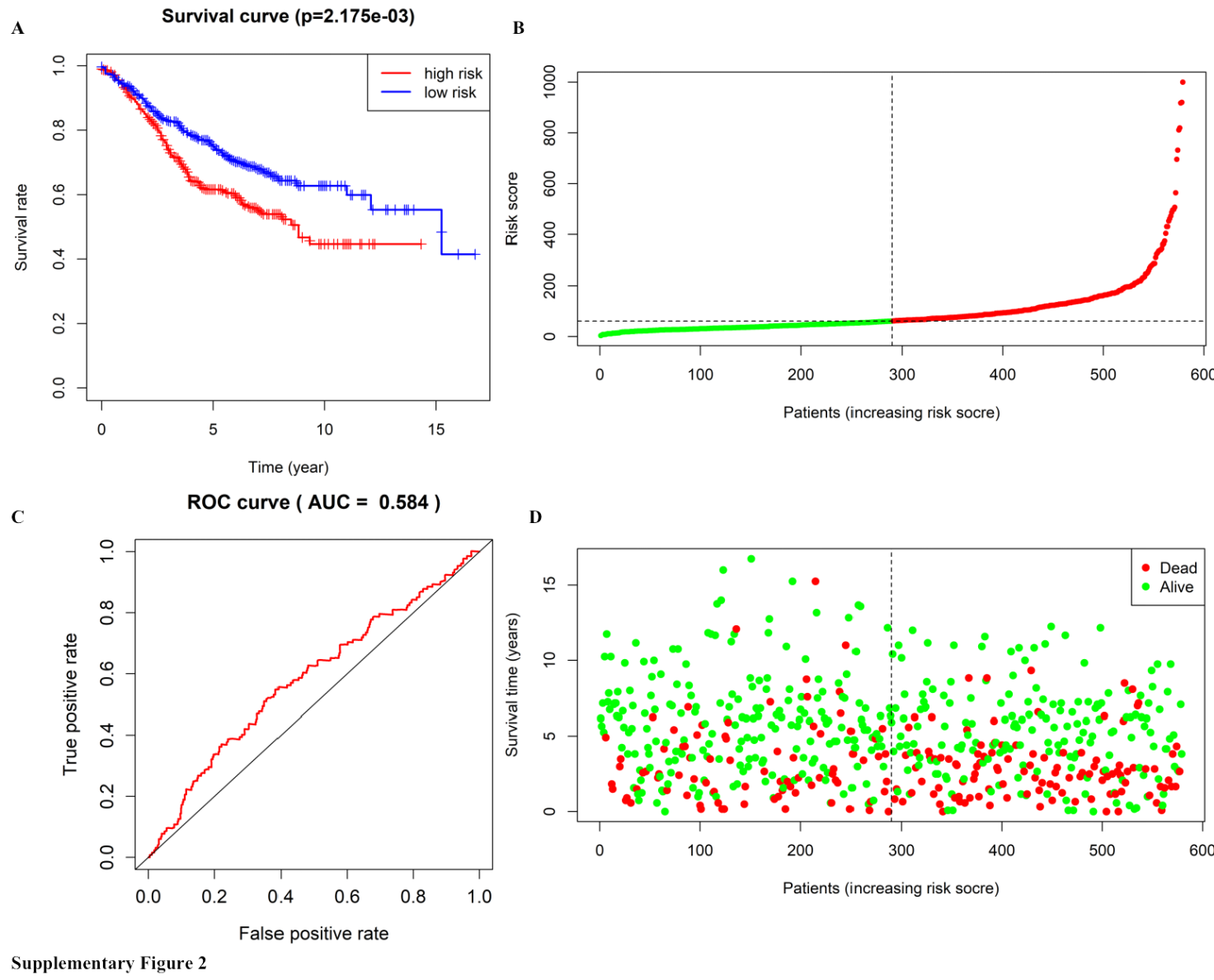


**Supplementary Figure 2.** Validation of the performance of the risk model using GEO dataset. (A) Kaplan-Meier (KM) analysis of OS based on the 8 FAM-related signature. (B) ROC analysis of the risk signature in predicting the OS. (C) Survival status distribution in high-risk group and low-risk group. (D) Risk score distribution of the high-risk group and low-risk group.
